# Smoking Is a Risk Factor for the Progression of Idiopathic Membranous Nephropathy

**DOI:** 10.1371/journal.pone.0100835

**Published:** 2014-06-25

**Authors:** Makoto Yamaguchi, Masahiko Ando, Ryohei Yamamoto, Shinichi Akiyama, Sawako Kato, Takayuki Katsuno, Tomoki Kosugi, Waichi Sato, Naotake Tsuboi, Yoshinari Yasuda, Masashi Mizuno, Yasuhiko Ito, Seiichi Matsuo, Shoichi Maruyama

**Affiliations:** 1 Department of Nephrology, Nagoya University Graduate School of Medicine, Nagoya, Japan; 2 Center for Advanced Medicine and Clinical Research, Nagoya University Hospital, Nagoya, Japan; 3 Department of Geriatric Medicine and Nephrology, Osaka University Graduate School of Medicine, Suita, Japan; The University of Auckland, New Zealand

## Abstract

**Background:**

Multiple studies have shown cigarette smoking to be a risk factor for chronic kidney disease. However, it is unknown whether smoking similarly increases the risk for progression of membranous nephropathy.

**Methods:**

This study used the Nagoya Nephrotic Syndrome Cohort Study (N-NSCS), including 171 patients with idiopathic membranous nephropathy (IMN) from 10 nephrology centers in Japan. The dose-response relationships between cigarette smoking and the outcomes were assessed by using multivariate Cox proportional hazards models adjusted for clinically relevant factors. The primary outcome was a 30% decline in the estimated glomerular filtration rate (eGFR). The secondary outcome was first complete remission (CR) of proteinuria.

**Results:**

During the observation period (median, 37 months; interquartile range, 16–71 months), 37 (21.6%) patients developed a 30% decline in eGFR and 2 (1.2%) progressed to ESRD. CR occurred in 103 (60.2%) patients. Multivariate Cox proportional hazards models revealed current smoking (adjusted hazard ratio [HR], 7.81 [95% confidence interval (CI), 3.17–19.7]), female sex (adjusted HR, 3.58 [95% CI, 1.87–8.00]), older age (adjusted HR, 1.71 [95% CI, 1.13–2.62] per 10 years), the number of cigarettes smoked daily (adjusted HR, 1.61 [95% CI, 1.23–2.09] per 10 cigarettes daily), and cumulative smoking of ≥40 pack-years (adjusted HR, 5.56 [95% CI, 2.17–14.6]) to be associated with a 30% decline in eGFR. However, smoking was not associated with CR.

**Conclusion:**

Smoking is a significant and dose-dependent risk factor for IMN progression. All patients with IMN who smoke should be encouraged to quit.

## Introduction

Membranous nephropathy (MN) is a very common cause of nephrotic syndrome in adults [1], [2]. Spontaneous remission occurs in 30–50% of patients, whereas another 30–50% experience progressive renal failure [3], [4]. Previously identified clinical predictors of poor renal survival include older age, male sex, elevated serum creatinine level at the time of diagnosis, and the severity of proteinuria at the time of disease onset and during follow-up [5]–[9]. However, most of these cannot be modified, and the long-term outcome is not unfavorable regardless: the 10-year renal survival ranges from 60 to 80% [10], [11]. The optimal management strategy for MN remains unclear.

It has recently become apparent that cigarette smoking, in addition to promoting cardiovascular disease (CVD), is an important independent renal risk factor [12]–[21]. However, these studies included patients with heterogeneous causes of CKD, such as diabetes, nephrosclerosis, and other diseases. Therefore, it was uncertain whether all kidney diseases were equally exacerbated by cigarette smoking. Elisabeth et al. reported in a nationwide population-based case-control study that the relationship between cigarette smoking and kidney impairment varied with the underlying kidney disease [22].

Yamamoto et al. reported in a large-scale retrospective cohort study that cigarette smoking was a key, dose-dependent prognostic factor in patients with IgA nephropathy [23]. The link between smoking and nephrotic syndrome has been investigated in only one case-control study of 80 patients with MN [24]. Although that study showed no relationship between smoking and MN, it was limited by the omission of two potentially significant independent variables, baseline proteinuria and kidney function at the time of study entry.

This study aimed to determine whether a history of smoking is an independent risk factor for the progression of MN and whether such risk is dose-dependent. This multicenter observational cohort study was organized as part of the Nagoya Nephrotic Syndrome Cohort Study (N-NSCS) based at 10 major nephrology centers in Nagoya, Japan.

## Subjects and Methods

### Study Population and Data Source

This cohort study included patients aged >18 years who had been diagnosed with membranous nephropathy (MN) on the basis of kidney biopsy between January 2003 and December 2012 at Nagoya University, Chubu Rosai Hospital, Japanese Red Cross Nagoya Daiichi Hospital, Tsushima City Hospital, Kasugai Municipal Hospital, Nagoya Kyoritsu Hospital, Anjo Kosei Hospital, Ichinomiya Municipal Hospital, Handa City Hospital, or Tosei General Hospital. Of the 272 such patients, we excluded patients with conditions generally considered to cause secondary MN, such as exposure to drugs associated with MN, diabetes mellitus, systemic lupus erythematosus, malignancy, or any other systemic disease known to be associated with secondary MN [25].

After exclusion of an additional 7 (3.9%) patients with missing data related to smoking status, 171 (62.9%) patients with idiopathic membranous nephropathy (IMN) were enrolled and followed up until September 2013. Clinically relevant factors at the baseline of those excluded (n = 7) and enrolled (n = 171) were not significantly different.

Our study was conducted by using linkable anonymous data set. No informed consent was obtained. The study protocol and consent procedure were approved by the ethics committees of Nagoya University, Chubu Rosai Hospital, Japanese Red Cross Nagoya Daiichi Hospital, Tsushima City Hospital, Kasugai Municipal Hospital, Nagoya Kyoritsu Hospital, Anjo Kosei Hospital, Ichinomiya Municipal Hospital, Handa City Hospital, and Tosei General Hospital Approval number:2012–0268.

### Data Collection

Data pertaining to the patients' clinical characteristics at baseline were collected retrospectively from their medical records. The time of kidney biopsy was used as the baseline if the patient had not received immunosuppressive therapy or had received immunosuppressive therapy only after kidney biopsy. In patients who had received immunosuppressive therapy before kidney biopsy, the time of the initiation of immunosuppressive treatment was used as the baseline. The clinical characteristics included age, sex, body mass index, systolic and diastolic blood pressure, serum total cholesterol level, serum creatinine level, glomerular filtration rate (GFR; estimated using the equation recently generated by the Japanese Society of Nephrology: eGFR [mL/min/1.73 m^2^] = 194×Scr^−1.094^×Age^−0.287^×0.739 [if female] [26]), serum albumin level, 24-hour urinary protein excretion or urinary protein-to-creatinine ratio, smoking status, use of any antihypertensive drugs, and initial use of corticosteroids and/or other immunosuppressive agents. Urinary protein excretion and eGFR data were collected from the medical record at each checkup in 1 to 3 month intervals.

We obtained detailed information about each patient's smoking status at the time of kidney biopsy, including the amount smoked (cigarettes/day), starting and stopping dates, and changes in use over time, from the medical records. The relationships between smoking and outcome measures were then studied for the following variables: (1) smoking status (all patients): never smoked, ex-smoker, or current smoker; (2) number of cigarettes smoked daily (current and ex-smokers); and (3) cumulative quantity smoked (current and ex-smokers): 0, 1–20, 21–39, or ≥40 pack-years (PY; 1 PY = 1 pack of 20 cigarettes/day ×1 year). Because we were most interested in whether a history of smoking was a risk factor for the progression of IMN, current smokers and ex-smokers were combined into a single group (current/ex-smokers).

The antihypertensive drugs used in this cohort were angiotensin-converting enzyme (ACE) inhibitors or angiotensin II receptor blockers (ARB), calcium channel blockers, β-blockers, and thiazides. The drugs used for therapeutic intervention, including the prescription of ACE inhibitors/ARB and corticosteroids or other immunosuppressive agents within 6 months of kidney biopsy, were also examined.

Nephrotic syndrome was defined as urinary protein excretion of ≥3.5 g/day (or a urinary protein/creatinine ratio of ≥3.5) and a serum albumin level of <3.0 mg/dL.

Complete remission (CR) of proteinuria was defined as urinary protein excretion of <0.3 g/day, a urinary protein/creatinine ratio of <0.3, and/or a negative/trace result for urinary protein on a dipstick test.

Partial remission (PR) of proteinuria was defined as urinary protein excretion of <3.5 g/day and a urinary protein/creatinine ratio of <3.5.

Relapse was defined as urinary protein excretion of ≥1.0 g/day, a urinary protein/creatinine ratio of ≥1.0, or a urinary protein dipstick result of ≥2+ on at least 2 occasions after achievement of CR.

The rate of decline in eGFR per year (mL/min per 1.73 m^2^/year) was determined by plotting the eGFR against the observation time.

The anonymous data set is available in the Table S4.

### Outcomes

The primary outcome was a 30% decline in eGFR before end-stage renal disease (ESRD). The secondary outcome was first CR.

The serum creatinine level was measured as required for each patient. Patients were followed up until September 2013 and censored at the time of death (if before ESRD or CR) or as of the last serum creatinine measurement before September 2013.

### Statistical Analysis

Differences in clinical characteristics between those who had never smoked and current-/ex-smokers were compared by using the Wilcoxon rank-sum test or Fisher's exact test. To identify predictors independently associated with each outcome, potential covariates were examined by using the log-rank test and/or univariate and multivariate Cox proportional hazards models. The proportional hazards assumption for covariates was tested by using scaled Schoenfeld residuals. For continuous variables, the Wilcoxon rank-sum test was performed to assess the significance of inter-group differences. Categorical variables were expressed as percentages and compared by using Fisher's exact test. The rate of survival without a 30% decline in eGFR and the cumulative probability of achieving a first CR were calculated by using the Kaplan-Meier method and the log-rank test. The trend in each outcome with respect to the cumulative quantity smoked was examined statistically by scoring never smoking as 0 and current-/ex-smokers with 1–20, 21–39, and ≥40 pack-years' exposure as 1, 2, and 3, respectively; the resulting scores were then included in the regression model. The level of statistical significance was set at *P*<0.05. All statistical analyses were performed by using JMP version 10.0.0 (SAS Institute, Cary, NC, USA; www.jmp.com) and STATA version 13.0 (STATA Corp, www.stata.com).

## Results

### Study participants and clinical characteristics

The present study included 171 patients with IMN, of whom 108 (63.2%) had never smoked, 28 (16.4%) were ex-smokers, and 35 (20.5%) were current smokers. The clinical characteristics of the two groups (those who had never smoked and current/ex-smokers) are summarized in Table 1.

**Table 1:** Clinical Characteristics of the 171 Patients with IMN.

	Never smoked	Smokers (Current/Ex-)	*P* value
Number	108	63 (35/28)	
Baseline characteristics			
Age (years)	66 (59–73)	63 (53–68)	0.012
Male [n (%)]	63 (58.3)	55 (87.3)	<0.001
Body mass index (kg/m^2^)	22.9 (21.1–24.7)	23.5 (22.3–26.2)	0.046
Systolic blood pressure (mmHg)	130 (120–145)	132 (121–143)	0.873
Diastolic blood pressure (mmHg)	76 (70–84)	79 (72–86)	0.156
Serum creatinine (mg/dL)	0.78 (0.66–1.00)	0.80 (0.71–1.00)	0.171
eGFR (mL/min/1.73 m^2^)	76 (58–92)	76 (63–86)	0.639
Serum albumin (g/dL)	2.6 (2.1–3.2)	2.4 (2.0–3.2)	0.518
Urinary protein (g/day)	3.9 (2.4–6.1)	4.8 (3.2–8.4)	0.025
Urinary protein >3.5 (g/day) [n (%)]	68 (63.0)	46 (73.0)	0.239
Total cholesterol (mg/dL)	283 (238–375)	295 (247–368)	0.617
Use of antihypertensive drugs [n (%)]	33 (30.6)	25 (39.7)	0.244
Smoking status			
Number of cigarettes smoked daily	NA	20 (20–30)	
Pack-years	NA	40 (24–49)	
Therapeutic interventions within 6 months after kidney biopsy			
ACE inhibitor or ARB therapy [n (%)]	65 (60.2)	48 (76.2)	0.044
Immunosuppressive treatment			0.413
No immunosuppressive agent	58 (53.7)	28 (44.4)	
Prednisolone [n (%)]	19 (17.6)	11 (17.5)	
Prednisolone + cyclosporine [n (%)]	31 (28.7)	24 (38.1)	
Outcomes			
30% reduction in eGFR [n (%)]	16 (14.8)	21 (33.3)	0.007
50% reduction in eGFR [n (%)]	6 (5.6)	11 (17.5)	0.017
Decline in eGFR (mL/min per 1.73 m^2^ per year)	2.42 (−1.44 to 6.91)	4.01 (0.51–8.78)	0.046
ESRD [n (%)]	1 (0.9)	1 (1.6)	1.000
Death [n (%)]	8 (7.4)	3 (4.8)	0.748
Remission			
Complete remission [n (%)]	63 (58.3)	40 (63.5)	0.522
Partial remission [n (%)]	96 (88.9)	54 (85.7)	0.631
Relapse [n (%)]	14 (16.1)	12 (26.7)	0.170
Observation period (months)	36 (15–71)	39 (18–74)	0.375

Median (interquartile range), Conversion factors for units: SCr in mg/dL to µmol/L, ×88.4; eGFR (mL/min/1.73 m^2^) = 194×Scr^−1.094^×Age^−0.287^×0.739 (if female), total cholesterol in mg/dL to mmol/L, ×0.02586.

Abbreviations: IMN, idiopathic membranous nephropathy; eGFR, estimated glomerular filtration rate; ACE inhibitor/ARB, angiotensin-converting enzyme inhibitor/angiotensin receptor blocker; SCr, serum creatinine; ESRD, end-stage renal disease; NA, not applicable.

The two groups differed significantly in age, proportion of males, body mass index, urinary protein excretion, and rate of therapeutic intervention with an ACE inhibitor or ARB within 6 months of kidney biopsy. At baseline, 114 patients (66.7%) had nephrotic syndrome as previously defined. The median follow-up period of those who had never-smoked, ex-smokers, and current-smokers was 36 (15–71), 33 (16–63), and 46 (18–87) months, respectively (*P* = 0.288).

We obtained the number of cigarettes smoked daily for 62 (98.4%) of the current/ex-smokers (1–10 cigarettes for 10 [16.1%], 11–20 cigarettes for 32 [51.6%], 21–30 cigarettes for 9 [14.5%], and 31–50 cigarettes for 11 [17.7%] patients) and the cumulative smoking dose for 60 (95.2%) of the current-/ex-smokers (≤20 pack-years for 11 [18.3%], 21–39 pack-years for 15 [25.0%], and ≥40 pack-years for 34 [56.7%] patients).

### Treatment during the observation period

An ACE inhibitor or ARB was newly prescribed within 6 months of kidney biopsy in 113 (66.1%) patients and was used by 156 (91.2%) by the end of follow-up. The enrolled patients were divided according to the treatment within 6 months of kidney biopsy into three groups: (1) the prednisolone group, comprising 30 patients (17.5%) who received prednisolone alone; (2) the cyclosporine group, comprising 55 patients (32.1%) who received prednisolone and cyclosporine; and (3) the supportive therapy group, comprising 86 patients (50.3%) who received neither prednisolone nor other immunosuppressive drugs. One patient in the cyclosporine group (0.6%) also received mizoribine. Most of the patients in the prednisolone group were prescribed prednisolone starting at 0.8–1.0 mg/kg/day orally and tapered according to the response to therapy, whereas most in the cyclosporine group started with cyclosporine at 1 to 2 mg/kg and prednisolone at 0.4–0.6 mg/kg. The cyclosporine dosage was adjusted to achieve the target whole-blood trough level, and the prednisolone dose was tapered according to the response to therapy. The time from renal biopsy to the initiation of immunosuppressive therapy was 0.7 months (interquartile range, 0.3–3.3 months). The duration of prednisolone use was 11 months (interquartile range, 8–25 months) in the prednisolone group and 14 months (interquartile range, 9–17 months) in the cyclosporine group.

### Outcome data

The median observation time for the entire cohort was 37 months (interquartile range, 15–72 months). In total, 37 (21.6%) patients developed a 30% decline in eGFR before ESRD, 17 (9.9%) developed a 50% decline in eGFR before ESRD, and 2 (1.2%) progressed to ESRD.

**Figure 1:**
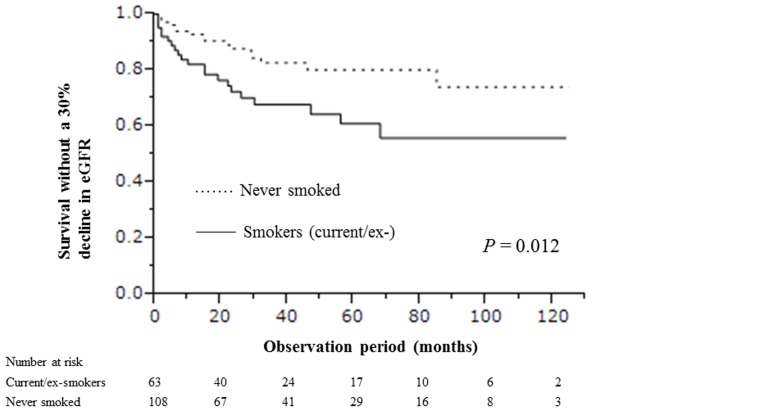
Time to 30% decline in eGFR in current/ex-smokers and those who never smoked.

The rates of avoiding a 30% decline in eGFR for 1, 5, and 10 years were 0.82 (95% confidence interval [CI], 0.71–0.90), 0.61 (95% CI, 0.46–0.74), and 0.56 (95% CI, 0.40–0.71), respectively, in current-/ex-smokers and 0.93 (95% CI, 0.86–0.97), 0.80 (95% CI, 0.69–0.88), and 0.74 (95% CI, 0.57–0.86) in those who had never smoked; therefore, current/ex-smokers were at higher risk for developing a 30% decline in eGFR (*P* = 0.012; Fig. 1).

The eGFR decreased significantly faster in current/ex-smokers than in those who had never smoked (4.01 mL/min per 1.73 m^2^ per year [interquartile range, 0.51–8.78 mL/min per 1.73 m^2^ per year] versus 2.42 mL/min per 1.73 m^2^ per year [interquartile range, −1.44 to 6.91 mL/min per 1.73 m^2^ per year]).

The eGFR did not improve by the last follow-up visit in any of the 37 patients who experienced a 30% decline in eGFR.

Meanwhile, 103 (60.2%) patients achieved CR of proteinuria after a median follow-up of 14 months (interquartile range, 6–25 months). The cumulative probabilities of achieving CR within 1, 5, and 10 years were 0.31 (95% CI, 0.20–0.44), 0.77 (95% CI, 0.62–0.87), and 0.81 (95% CI, 0.65–0.90) in current-/ex-smokers and 0.43 (95% CI, 0.33–0.54), 0.80 (95% CI, 0.67–0.88), and 0.81 (95% CI, 0.65–0.92) in those who had never smoked. Therefore, smoking was not associated with the achievement of CR (*P* = 0.570).

Among the patients who achieved a first remission, 26 (25.2%) relapsed at least once.

No patient progressed to ESRD before developing a 30% decline in eGFR.

Eleven (6.4%) patients died during follow-up, one due to infection, and one each to acute subdural hematoma, traffic accident, sudden death, and intestinal bleeding. Malignancy occurred in 5 (2.9%) patients; the diagnoses were esophageal cancer, stomach cancer, colon cancer, prostate cancer, and malignant lymphoma in one patient each.

### Predictors of a 30% decline in eGFR

Univariate Cox proportional hazards models revealed that older age, female sex, initial use of cyclosporine combination therapy, and current smoking were significantly associated with the primary outcome (Table 2). Adjustment for clinically relevant factors revealed current smoking (adjusted hazard ratio [HR], 7.81 [95% CI, 3.17–19.7], *P*<0.001), female sex (adjusted HR, 3.58 [95% CI, 1.87–8.00], *P* = 0.002), older age (adjusted HR, 1.71 [95% CI, 1.13–2.62], per 10 years, *P* = 0.010), and use of cyclosporine in combination with prednisolone (adjusted HR, 2.67 [95% CI, 1.08–6.59], *P* = 0.034) to be significant predictors of the primary outcome.

**Table 2:** Predictors of a 30% decline in eGFR.

	Univariate model		Multivariate model	
	HR (95% CI)	*P* value	HR (95% CI)	*P* value
Age (per 10 years)	1.47 (1.07–2.07)	0.018	1.71 (1.13–2.62)	0.010
Male (versus female)	0.43 (0.22–0.83)	0.012	0.28 (0.13–0.63)	0.002
Systolic blood pressure (per 10 mmHg)	1.06 (0.90–1.23)	0.455	1.07 (0.86–1.32)	0.546
Diastolic blood pressure (per 10 mmHg)	1.20 (0.94–1.53)	0.142	1.08 (0.77–1.52)	0.660
Serum albumin (per 1.0 g/dL)	0.80 (0.51–1.24)	0.317	1.32 (0.73–2.36)	0.359
Serum creatinine (per 1.0 mg/dL)	0.67 (0.18–1.98)	0.501	0.90 (0.19–3.13)	0.885
Urinary protein excretion (per 1.0 g/day)	1.03 (0.94–1.12)	0.519	1.02 (0.91–1.13)	0.673
Therapeutic interventions within 6 months after kidney biopsy				
ACE inhibitor or ARB therapy	0.72 (0.26–2.99)	0.598	0.90 (0.29–3.96)	0.874
Immunosuppressive treatment				
No immunosuppressive agent	Reference		Reference	
Prednisolone	0.84 (0.24–2.33)	0.758	1.30 (0.33–4.21)	0.687
Prednisolone + cyclosporine	2.18 (1.09–4.42)	0.027	2.67 (1.08–6.59)	0.034
Smokers (Current/Ex-)	2.24 (1.17–4.35)	0.015	4.00 (1.87–8.79)	<0.001
Ex-smokers	1.68 (0.64–3.93)	0.273	2.23 (0.79–5.79)	0.127
Current smokers	2.68 (1.29–5.52)	0.009	7.81 (3.17–19.7)	<0.001

HR, hazard ratio; CI, confidence interval.

Data are the HR, 95% CI, and *P* value from Cox proportional hazard regression analyses.

“Never smoked” was used as the reference category.

Adjusted for baseline characteristics (age, sex, systolic/diastolic pressure, serum creatinine level, urinary protein, use of ACE inhibitor or ARB within 6 months after kidney biopsy, and immunosuppressive therapy within 6 months after kidney biopsy).

Abbreviations: IMN, idiopathic membranous nephropathy; ACE, angiotensin-converting enzyme; ARB, angiotensin receptor blocker.

No interactive effect on the primary outcome was detected between use of cyclosporine and smoking (adjusted HR, 1.16 [95% CI, 0.61–2.24], *P* = 0.644).

To confirm the robustness of our results, we similarly assessed predictors of a 50% decline in eGFR and found that current smoking (adjusted HR, 9.85 [95% CI, 2.68–41.0], *P*<0.001) and female sex (adjusted HR, 4.20 [95% CI, 1.19–15.3], *P* = 0.027) were also significant predictors of this outcome (Table S1).

### Dose-dependent association between smoking and a 30% decline in eGFR

Table 3 shows that the total smoking dose had a strong independent effect on the risk of renal progression. Both univariate and multivariate Cox proportional hazards models showed a significant association between the number of cigarettes per day and the primary outcome (adjusted HR, 1.61 [95% CI, 1.23–2.09], *P*<0.001, multivariate model 1). Cumulative smoking of ≥40 pack-years was also significantly associated with the primary outcome (adjusted HR, 5.56 [95% CI, 2.17–14.6], *P*<0.001, multivariate model 2). These results suggested that the risk of renal progression increased almost linearly with the number of cigarettes smoked.

**Table 3:** Influence of smoking dose on the risk of a 30% decline in eGFR.

	Univariate model		Multivariate model	
	HR (95% CI)	*P* value	HR (95% CI)	*P* value
Model 1				
No. of cigarettes (/10/d)	1.32 (1.05–1.63)	0.021	1.61 (1.23–2.09)	<0.001
Model 2				
1–20 pack-years	1.07 (0.17–3.75)	0.933	1.36 (0.20–5.94)	0.715
21–39 pack-years	1.64 (0.47–4.49)	0.399	3.51 (0.87–12.5)	0.076
≥40 pack-years	2.61 (1.21–5.48)	0.016	5.56 (2.17–14.6)	<0.001
Test for trend		0.015		<0.001

HR, hazard ratio; CI, confidence interval.

Data are the HR, 95% CI, and *P* value from Cox proportional hazard regression analyses.

“Never smoked” was used as the reference category. Models 1 and 2 are based on data from 168 patients because the number of cigarettes was missing for 1 current and 2 ex-smokers. Adjusted for baseline characteristics (age, sex, systolic/diastolic pressure, serum creatinine level, urinary protein, use of angiotensin-converting enzyme inhibitor or angiotensin receptor blocker within 6 months after kidney biopsy, and immunosuppressive therapy within 6 months after kidney biopsy).

### Predictors of first complete remission

Univariate Cox proportional hazards models revealed a lower serum creatinine level, initial use of prednisolone monotherapy, and initial use of cyclosporine in combination with prednisolone to be significantly associated with first CR of proteinuria (Table S2). After adjustment for clinically relevant factors, the initial use of prednisolone monotherapy (adjusted HR, 2.15 [95% CI, 1.17–3.84], *P* = 0.015) and cyclosporine in combination with prednisolone (adjusted HR, 2.90 [95% CI, 1.68–5.00], *P*<0.001) remained significant predictors of first CR of proteinuria. Neither a history of smoking (Table S2) nor the total smoking dose (Table S3) was associated with CR.

## Discussion

We found that smoking increased the risk of renal dysfunction in Japanese patients with IMN in a dose-dependent manner, although former smoking did not appear to be a risk factor. These results highlight the clinical importance of smoking cessation in the treatment of patients with IMN.

Several cohort studies in the general population have suggested a dose-dependent association between smoking and CKD. The Cardiovascular Health Study identified smoking as a risk factor related to CKD progression in 4142 U.S. participants aged >65 years but did not include proteinuria as an independent variable [12]. The NHANRS II study followed 9082 middle-aged (49.3±13.3 years) U.S. participants for 13.2 years and showed a higher adjusted risk of end-stage kidney disease among those smoking >20 cigarettes per day. Baggio et al. studied 2981 elderly subjects from Italy and found smoking ≥21 cigarettes per day to be a very strong risk factor for pathological loss of kidney function [17]. Furthermore, the HUNT II study of 65,589 Norwegian participants revealed that the risk increased significantly as the cumulative amount of smoking (pack-years) increased; however, the risk significantly decreased as the years elapsed since smoking cessation increased among male ex-smokers aged <70 years [27]. Yamagata reported in a 10-year follow-up study of 123,764 Japanese participants aged ≥40 years who received community-based annual examinations that current smoking increased the risk of developing proteinuria, but that study did not evaluate the dose-dependency of the relationship between smoking and CKD [21]. These reports collectively identify smoking as a risk factor for the progression of CKD in the general population. Our present results were consistent with this literature, particularly the HUNT II study, in which current/ex-smokers reporting larger cumulative numbers of cigarettes smoked were at significantly higher risk of renal progression than were those who had never smoked.

Only a few studies of primary kidney disease have found a dose-dependent association between cigarette smoking and progression of primary glomerulonephritis. Yamamoto et al. identified cigarette smoking as a key dose-dependent prognostic factor for IgA nephropathy in a multivariate model. There has been only one case-control study addressing the relationship between smoking and nephrotic syndrome, which revealed no relationship between smoking and kidney progression in patients with MN [24]. However, that study failed to include baseline proteinuria and kidney function at study entry as independent variables. In contrast, we included those variables and identified smoking as an important dose-dependent prognostic factor in patients with IMN after adjustment for relevant confounders.

The present study identified immunosuppressive therapy as both a risk factor for renal progression (although urinary protein excretion was greater in such patients than in those who initially received supportive therapy) and the most significant independent predictor of achievement of CR. These results may, however, reflect indication bias, for which the present retrospective cohort study did not control. Furthermore, we should consider that unmeasured factors associated with treatment may not be included in the model.

Combination therapy with cyclosporine and prednisolone was frequently used for treatment of IMN in our cohort. Cattran et al. demonstrated that cyclosporine significantly decreased the rate of renal progression and reduced proteinuria in patients with IMN at high risk for progression and persistent nephrotic-range proteinuria [28], [29]. Alexopoulos et al. also found that cyclosporine with or without corticosteroids effectively induced remission in the majority of nephrotic patients with IMN and well-preserved renal function [30]. Based on these findings, we often use cyclosporine in combination with corticosteroids for first-line treatment of patients with IMN. Although the 2012 Kidney Disease Improving Global Outcomes (KDIGO) clinical practice guideline for IMN recommends a cytotoxic agent (cyclophosphamide) for patients at high risk of progression [31], no patient in our study was treated with cyclophosphamide. This should be considered when interpreting our results.

Interestingly, our study identified female sex as an independent risk factor after adjustment for relevant clinical factors. Previous studies had shown male sex to be associated with a higher risk of developing ESRD [5]–[9]. In contrast, female sex has generally been associated with a relatively benign course [32]. However, these studies did not include smoking status as an independent variable, which may have caused overestimation of the relationship between male sex and risk of renal progression. Furthermore, Mina Yu reported in a cross-sectional study that menopause was an independent risk factor for CKD because of the accompanying loss of possibly cardioprotective estradiol production [33]. Previous studies of membranous nephropathy have generally enrolled younger patients than our study, suggesting that our study may have included a relatively large number of postmenopausal patients. Therefore, membranous nephropathy may lead to more rapid decline in GFR in postmenopausal women, who are already in an unfavorable hormonal state (i.e., estrogen deficient) with respect to the kidney. The generalizability of our results should be confirmed in another cohort study.

Although the exact mechanisms of the nephrotoxic effects of smoking are poorly understood, we can present some potential explanations of the relationship between smoking and CKD onset and progression. First, nephrotic syndrome increases oxidative stress by impairing antioxidant pathways [34]. The addition of smoking-induced oxidative stress to nephrotic syndrome-associated oxidative stress may accelerate renal progression [35]–[38]. Second, ex vivo studies have suggested that nicotine could promote mesangial cell proliferation and increase production of critical molecules involved in extracellular matrix production [39], [40]. Third, smoking (or nicotine) could increase the plasma endothelin level [41], which has been shown to correlate with effective renal plasma flow in smokers [42]. Furthermore, Alves et al. reported that smoking aggravates the cyclosporine-induced impairment of GFR and that smoking in combination with cyclosporine induced periglomerular structural lesions and oxidative stress in a rat model of cyclosporine nephrotoxicity [43]. However, our results showed no interactive effect on renal function between use of cyclosporine and smoking. The external validity of the result should be confirmed in another study.

It is also possible that smoking might relate to medication adherence in some other way. A cross-sectional study showed that current smokers were less compliant with medication use than nonsmokers [44]. Therefore, we should consider the possibility of medication adherence in smokers. Moreover, we are aware that MN can itself have extra-renal associations and these should be considered for their possible relationship with smoking.

Our study has some limitations that must be discussed. First, underreporting of smoking and changes in smoking habits during the follow-up period can cause misclassification and tend to overestimate the effect of smoking on renal progression. Second, we did not address the relationship between smoking cessation and renal progression because our sample size was insufficient to evaluate it. Third, the patients in this study may not be representative of patients with IMN in other countries: different consequences of smoking have been reported in different populations [45]. We therefore advise caution when interpreting and generalizing our results.

Allowing for these methodological issues, our study has several advantages: it is one of the largest multicenter adult MN cohorts in Japan ever reported and is also, to the best of our knowledge, the first description of a relationship between smoking and renal dysfunction in patients with IMN.

In conclusion, our retrospective cohort study in patients with IMN showed cigarette smoking to be a key, dose-dependent predictor of IMN progression. All patients with IMN who smoke should be encouraged to quit.

## Supporting Information

Table S1 **Predictors of a 50% decline in eGFR.**
(DOC)

Table S2 **Predictors of first CR.**
(DOCX)

Table S3 **Influence of smoking dose on first CR.**
(DOCX)

Table S4 **The anonymous data set of 171 patients with IMN.**
(XLSX)
