# Supplementary material for: Smoking Is a Risk Factor for the Progression of Idiopathic Membranous Nephropathy
Source: PLoS One. 2014 Jun 25;9(6):e100835. doi: 10.1371/journal.pone.0100835 (PMC4071015; doi:10.1371/journal.pone.0100835)
Supplement: Table S1 — Predictors of a 50% decline in eGFR. (DOC) [file pone.0100835.s001.doc]

**Table S1. Predictors of a 50% decline in eGFR**

|  | **Univariate model** | | **Multivariate model** | |
| --- | --- | --- | --- | --- |
|  | **HR (95% CI)** | ***P* value** | **HR (95% CI)** | ***P* value** |
| Age (per 10 years) | 1.10 (0.72–1.77) | 0.673 | 1.05 (0.57–1.98) | 0.878 |
| Male (versus female) | 0.59 (0.23–1.63) | 0.296 | 0.24 (0.07–0.84) | 0.027 |
| Systolic blood pressure (per 10 mmHg) | 1.06 (0.83–1.32) | 0.692 | 0.92 (0.66–1.25) | 0.612 |
| Diastolic blood pressure (per 10 mmHg) | 1.42 (1.00–1.98) | 0.049 | 1.50 (0.89–2.61) | 0.130 |
| Serum albumin (per 1.0 g/dL) | 0.57 (0.28–1.10) | 0.093 | 0.85 (0.34–1.96) | 0.708 |
| Serum creatinine (per 1.0 mg/dL) | 2.20 (0.50–6.60) | 0.265 | 5.02 (0.91–21.5) | 0.062 |
| Urinary protein excretion (per 1.0 g/day) | 1.04 (0.91–1.17) | 0.512 | 0.91 (0.73–1.08) | 0.291 |
| Therapeutic interventions within 6 months after kidney biopsy |  |  |  |  |
| ACE inhibitor or ARB therapy | 0.38 (0.10–2.45) | 0.260 | 0.44 (0.10–3.11) | 0.364 |
| Immunosuppressive treatment |  |  |  |  |
| No immunosuppressive agent | Reference |  | Reference |  |
| Prednisolone | 2.76 (0.68–10.5) | 0.147 | 2.76 (0.58–12.6) | 0.196 |
| Prednisolone + cyclosporine | 2.93 (0.97–9.76) | 0.057 | 2.50 (0.60–10.6) | 0.205 |
| Smokers (Current/Ex-) | 2.98 (1.13–8.65) | 0.027 | 5.35 (1.65–20.5) | 0.005 |
| Ex-smokers | 1.89 (0.40–7.18) | 0.387 | 2.57 (0.47–12.4) | 0.257 |
| Current smokers | 3.79 (1.32–11.5) | 0.014 | 9.85 (2.68–41.0) | <0.001 |

HR, hazard ratio; CI, confidence interval

Data are the HR, 95% CI, and *P* value from Cox proportional hazard regression analyses.

“Never smoked” was used as the reference category.

Adjusted for baseline characteristics (age, sex, systolic/diastolic pressure, serum creatinine level, urinary protein, use of ACE inhibitor or ARB within 6 months after kidney biopsy, and immunosuppressive therapy within 6 months after kidney biopsy).

Abbreviations: IMN, idiopathic membranous nephropathy; ACE, angiotensin-converting enzyme; ARB, angiotensin receptor blocker
