# Supplementary material for: Smoking Is a Risk Factor for the Progression of Idiopathic Membranous Nephropathy
Source: PLoS One. 2014 Jun 25;9(6):e100835. doi: 10.1371/journal.pone.0100835 (PMC4071015; doi:10.1371/journal.pone.0100835)
Supplement: Table S2 — Predictors of first CR. (DOCX) [file pone.0100835.s002.docx]

**Table S2. Predictors of first CR**

|  | **Univariate model** | | **Multivariate model** | |
| --- | --- | --- | --- | --- |
|  | **HR (95% CI)** | ***P* value** | **HR (95% CI)** | ***P* value** |
| Age (per 10 years) | 0.99 (0.83–1.19) | 0.886 | 1.01 (0.81–1.25) | 0.759 |
| Male (versus female) | 0.69 (0.46–1.04) | 0.075 | 0.66 (0.40–1.09) | 0.106 |
| Systolic blood pressure (per 10 mmHg) | 1.00 (0.91–1.09) | 0.959 | 1.11 (0.96–1.27) | 0.136 |
| Diastolic blood pressure (per 10 mmHg) | 0.98 (0.84–1.12) | 0.725 | 0.91 (0.74–1.13) | 0.354 |
| Serum albumin (per 1.0 g/dL) | 0.86 (0.67–1.11) | 0.249 | 1.16 (0.82–1.63) | 0.351 |
| Serum creatinine (per 1.0 mg/dL) | 0.47 (0.21–0.96) | 0.037 | 0.52 (0.21–1.14) | 0.107 |
| Urinary protein excretion (per 1.0 g/day) | 1.01 (0.95–1.06) | 0.730 | 0.99 (0.93–1.06) | 0.966 |
| Therapeutic interventions within 6 months after kidney biopsy |  |  |  |  |
| ACE inhibitor or ARB therapy | 0.66 (0.44–1.02) | 0.061 | 0.72 (0.46–1.13) | 0.152 |
| Immunosuppressive treatment |  |  |  |  |
| No immunosuppressive agent | Reference |  | Reference |  |
| Prednisolone | 1.76 (1.00–2.96) | 0.048 | 2.15 (1.17–3.84) | 0.015 |
| Prednisolone + cyclosporine | 2.18 (1.41–3.36) | <0.001 | 2.90 (1.68–5.00) | <0.001 |
| Smokers (Current/Ex-) | 0.91 (0.61–1.34) | 0.627 | 1.03 (0.66–1.60) | 0.880 |
| Ex-smokers | 1.04 (0.59–1.74) | 0.884 | 1.12 (0.62–1.94) | 0.693 |
| Current smokers | 0.81 (0.49–1.29) | 0.383 | 1.02 (0.58–1.73) | 0.952 |

HR, hazard ratio; CI, confidence interval

Data are the HR, 95% CI, and *P* value from Cox proportional hazard regression analyses.

“Never smoked” was used as the reference category.

Adjusted for baseline characteristics (age, sex, systolic/diastolic pressure, serum creatinine level, urinary protein, use of ACE inhibitor or ARB within 6 months after kidney biopsy, and immunosuppressive therapy within 6 months after kidney biopsy).

Abbreviations: CR, complete remission; ACE, angiotensin-converting enzyme; ARB, angiotensin receptor blocker
