# Supplementary material for: Smoking Is a Risk Factor for the Progression of Idiopathic Membranous Nephropathy
Source: PLoS One. 2014 Jun 25;9(6):e100835. doi: 10.1371/journal.pone.0100835 (PMC4071015; doi:10.1371/journal.pone.0100835)
Supplement: Table S3 — Influence of smoking dose on first CR. (DOCX) [file pone.0100835.s003.docx]

**Table S3. Influence of smoking dose on first CR**

|  | **Univariate model** |  | **Multivariate model** | |
| --- | --- | --- | --- | --- |
|  | **HR (95% CI)** | ***P* value** | **HR (95% CI)** | ***P* value** |
| Model 1 |  |  |  |  |
| No. of cigarettes (/10/d) | 0.93 (0.79–1.08) | 0.342 | 1.01 (0.84–1.21) | 0.895 |
| Model 2 |  |  |  |  |
| 1–20 pack-years | 1.73 (0.75–2.92) | 0.153 | 1.54 (0.67–3.17) | 0.288 |
| 21–39 pack-years | 0.97 (0.50–1.74) | 0.929 | 1.07 (0.50–2.08) | 0.856 |
| ≥40 pack-years | 0.69 (0.39–1.14) | 0.153 | 0.93 (0.50–1.66) | 0.814 |
| Test for trend |  | 0.215 |  | 0.979 |

HR, hazard ratio; CI, confidence interval

Data are the HR, 95% CI, and *P* value from Cox proportional hazard regression analyses.

“Never smoked” was used as the reference category. Models 1 and 2 are based on data from 168 patients because the number of cigarettes was missing for 1 current and 2 ex-smokers. Adjusted for baseline characteristics (age, sex, systolic/diastolic pressure, serum creatinine level, urinary protein, use of ACE inhibitor or ARB within 6 months after kidney biopsy, and immunosuppressive therapy within 6 months after kidney biopsy).

Abbreviations: CR, complete remission; ACE, angiotensin-converting enzyme; ARB, angiotensin receptor blocker
